# A Randomized Pilot Study of Time-Restricted Eating Shows Minimal Microbiome Changes

**DOI:** 10.3390/nu17010185

**Published:** 2025-01-04

**Authors:** Abigail J. Johnson, Alison Alvear, Dan Knights, Lisa S. Chow, Anne E. Bantle

**Affiliations:** 1 Division of Epidemiology and Community Health, School of Public Health, University of Minnesota, Minneapolis, MN 55455, USA; 2 Division of Diabetes, Endocrinology and Metabolism, Department of Medicine, University of Minnesota, Minneapolis, MN 55455, USAchow0007@umn.edu (L.S.C.); 3 Department of Computer Science & Engineering, University of Minnesota, Minneapolis, MN 55455, USA

**Keywords:** obesity, time-restricted eating, gut microbiome, fasting, weight loss

## Abstract

Objective: TRE is an emerging approach in obesity treatment, yet there is limited data on how it influences gut microbiome composition in humans. Our objective was to characterize the gut microbiome of human participants before and after a TRE intervention. This is a secondary analysis of a previously published clinical trial examining the effects of time-restricted eating (TRE). Methods: In a previously published, 12-week randomized controlled trial, Chow et al. evaluated the effects of an 8-h TRE intervention on body composition in human participants. Chow et al. demonstrated significant reductions in weight, lean mass, and visceral fat in the TRE group compared to those following time-unrestricted eating (non-TRE). Stool samples were collected by a subset of those participants using home kits at both baseline and post-intervention for shotgun metagenomic sequencing for this secondary analysis. Microbiome community composition was compared before and after intervention as alpha and beta diversity. Results: Sixteen participants provided stool samples (eight in the TRE group and eight in the non-TRE group). Stool samples were collected from all participants at at least one time point, but both pre- and post-treatment samples were available from only five participants who completed both baseline and post-treatment collections. In alignment with the findings of Chow et al., the participants in the TRE group of the secondary analysis who collected microbiome sample(s) successfully reduced their eating window from an average of 15.3 ± 0.8 h at baseline to 9.3 ± 1.7 h during the intervention (mean ± SD, *p* < 0.001) and the non-TRE group’s eating window remained unchanged. While the TRE group lost weight and visceral fat mass, no effect of the TRE intervention was observed on alpha diversity (Shannon index, Simpson index, and number of taxa, linear mixed models), beta diversity (Bray–Curtis, PERMANOVA), even after controlling for weight and visceral fat changes. Conclusions: Our analysis did not detect any significant differences in gut microbiome composition or diversity indices between participants undergoing a TRE intervention and those in the control group. The study’s findings are limited by a small sample size, short duration, and the collection of stool samples at only two time points. Future studies with larger sample sizes, longer durations, and more frequent sampling, and collection of detailed dietary data are needed to better understand the relationship between TRE and gut microbiome dynamics.

## 1. Introduction

Effective strategies for the prevention and treatment of obesity are urgently needed. Lifestyle interventions, particularly those involving dietary changes to promote weight loss, remain at the forefront of obesity management. Although various dietary patterns combined with caloric restriction have been shown to be effective for weight loss [1], many individuals continue to struggle with achieving long-term weight loss.

Time-restricted eating (TRE) is a dietary strategy that limits the daily window during which an individual is allowed to eat. There are various implementations of TRE such as alternate day fasting or 5 days of fasting and 2 days of eating. In the research literature, the most common implementation of TRE is daily fasting with a reduced eating window of 8–10 h. Unlike traditional calorie-restricted diets, TRE permits individuals to select foods based on personal preference, without setting specific caloric limits. Many studies have suggested that TRE can lead to weight loss and other metabolic benefits [2,3], though not all clinical trials conducted in humans have shown consistent results [4,5]. The simplicity of TRE—focusing on when to eat rather than what or how much to eat—makes it an appealing option for obesity treatment, offering a contrast to more traditional weight-loss diets that require greater patient tracking burden.

Findings are mixed regarding the effect of TRE on the human gut microbiome. While diet composition is a key driver of microbiome changes, emerging evidence suggests that the timing of food intake, independent of dietary content, can also influence the gut microbiome. For example, studies in mice have shown that TRE restores diurnal fluctuations in gut microbiome composition that are typically suppressed by diet-induced obesity, even when the diet remains unchanged [6]. In humans, Zeb et al. reported shifts in gut microbial composition following a TRE intervention, including increased abundance of *Prevotellaceae* and *Bacteroideaceae*, without instruction to alter diet composition [7]. Additionally, fasting during Ramadan, which involves a restricted eating window with similarities to TRE, has been associated with increased microbial diversity and enrichment of metabolically favorable microbial taxa [8]. These findings suggest that the timing of food intake, rather than the content of the diet, may play a critical role in shaping the gut microbiome.

Chow et al., conducted a randomized controlled trial involving an 8-h TRE intervention in human participants over 12 weeks and demonstrated reductions in weight, lean mass, and visceral fat compared to a control group [9]. Here, we perform an exploratory, secondary analysis of stool samples collected during this trial to assess changes in the gut microbiome following the TRE intervention, compared to baseline and a non-TRE control group.

## 2. Methods

This study is a secondary analysis of a 12-week randomized controlled trial evaluating an 8-h TRE intervention in human participants with obesity [9]. The protocol was approved by the University of Minnesota Institutional Review Board (project identification code 1701M06001) on 21 March 2017. All participants provided written informed consent prior to their participation. The study was registered at ClinicalTrials.gov (identification number NCT03129581). Detailed descriptions of the study protocol and data collection procedures have been previously published [9].

### 2.1. Study Design and Participants

This randomized controlled, unblinded study assigned participants in a 1:1 ratio to either the TRE or time-unrestricted eating (non-TRE) group for a duration of 12 weeks. Participants were 18–65 years of age, with a body mass index (BMI) ≥ 25 kg/m^2^, and were recruited from the University of Minnesota and the surrounding community. Additional inclusion criteria included having a stable sleep and work schedule (with consistent bedtimes and wake times within a 2-h window for at least 6 out of 7 days per week and no more than 4 h of variance on the seventh day), possession of a smartphone for logging meal timing, and the ability to provide informed consent. Exclusion criteria included current or anticipated pregnancy, nursing, or the presence of significant medical conditions, such as diabetes mellitus or cardiovascular disease.

Enrollment began in October 2017 and was completed by September 2018, when the prespecified recruitment goals were met. A pre-study power calculation determined that 20 participants total were needed to detect a minimum difference of 1.33 SD in body weight, with a two-sided α (false-positive rate) of 0.05 and power of 80% [9]. The study was not powered for the exploratory microbiome analysis pilot study. All participant follow-up was completed by December 2018. After initiation of the original study, stool collection was incorporated into the protocol; only participants who provided stool samples at baseline or post-intervention were included in this secondary analysis. Study data were collected and managed using REDCap electronic data capture tools, hosted at the University of Minnesota [10,11].

### 2.2. Tracking Meal Timing

After providing informed consent and completing a screening visit, participants were trained to use a smartphone-based application (myCircadianClock, mCC; Dr. Satchin Panda’s Lab, Salk Institute, La Jolla, CA, USA, circadian@mycircadianclock.org) to log the timing of their food intake [12]. They were instructed to take photos of each meal for at least one week using the app. Participants who used the mCC application for more than 80% of the time and whose profiles indicated an eating window exceeding 14 h per day were eligible to continue in the study. These participants were then randomly assigned to either the TRE group, where they were instructed to limit their eating to a self-selected 8-h window each day, or to the non-TRE group, which maintained unrestricted eating times. There were no restrictions on the types or amounts of food consumed for either group. All participants continued to use the mCC application to document their food and beverage intake throughout the study. Compliance was monitored weekly through remote tracking of app data. Participants received weekly feedback from the study team via text, email, or phone to encourage adherence to the assigned eating window (TRE group) and food logging (both groups).

### 2.3. Outcome Measures

The primary outcome of this secondary analysis was the change in gut microbiome composition, as assessed by alpha and beta diversity, between baseline and post-intervention. In the original trial, the primary outcome was the change in weight from pre- to post-intervention. Additional outcome measures, assessed at both baseline and post-intervention, included body composition (measured by dual X-ray absorptiometry [DXA]), blood pressure, fasting lipid profile, hemoglobin A1c (HbA1c), 2-h glucose levels during a 75 g oral glucose tolerance test (OGTT), glycemic variability (measured using a continuous glucose monitoring system [CGMS]), and physical activity levels. Study participants wore the FreeStyle Libre Pro (Abbott, Chicago, IL, USA) CGMS and the ActiGraph Link sensor (ActiGraph, Pensacola, FL, USA) for up to 14 days at both baseline and the end of the intervention. Results from these measures have been previously published [9].

### 2.4. Fecal Sample Collection

Participants were asked to submit stool samples at baseline and at the end of the intervention period. Home collection kits were provided, along with written and verbal instructions on the collection method. Stool samples were collected by participants at home using a Feces Catcher (Tag Hemi VOF, Zeijen, The Netherlands). A pea-sized amount of stool was transferred with a CultureSwab EZ sterile swab (Thermo Fisher Scientific, Waltham, MA, USA) into a 2.0 mL cryovial containing 1.0 mL of RNA*later* Stabilization Solution (Thermo Fisher Scientific, Waltham, MA, USA). The contents were thoroughly mixed and stored at room temperature until returned to study staff, either in person or by mail (within 7 days). Upon receipt, specimens were immediately stored at −80 °C in a freezer until the study’s completion. This collection method has been previously used for similar microbiome studies [13].

### 2.5. Microbiome Sequencing and Bioinformatics

Stool samples were submitted as a single batch to the University of Minnesota Genomics Center for DNA extraction, amplification, and sequencing. DNA was extracted using the Qiagen PowerSoil DNA extraction kit (Qiagen, Hilden, Germany), followed by a clean-up step using aZymo kit (Zymo Research, Irvine, CA, USA). Shotgun DNA sequencing was performed on the Illumina HiSeq platform (Illumina, San Diego, CA, USA). The mean sequencing depth for each sample before processing was 4,912,870 sequences. The resulting data were processed using the default settings of the Shi7 pipeline, as previously published [14]. Raw sequences were trimmed and filtered for quality, and paired sequences were stitched using Shi7. Quality-controlled sequences were aligned to the GTDB Genome Taxonomy Database (https://gtdb.ecogenomic.org) using BURST [15]. After alignment, a mean of 1,075,565 ± 233,856 reads per sample were assigned a taxonomic classification. Rare taxa, present in less than 10% of all samples, were dropped, as were taxa that had an average relative abundance less than or equal to 0.05%. Diversity metrics were calculated from the species-level median-scaled relative abundance data.

### 2.6. Statistical Analysis

Descriptive statistics were calculated using Fisher’s exact test, Student’s *t*-test, or the Welch Two-Sample *t*-test, as appropriate. The effect of the TRE intervention on alpha diversity (Shannon, Simpson, and number of taxa) were modeled using linear mixed models, implemented with the *lmer* function from the *lmerTest* package in *R* [16]. *p*-values were calculated using the *emmeans* function in *R* [17]. The effect of the TRE intervention on beta diversity was tested using permutational multivariate analysis of variance (PERMANOVA) using the *adonis* and *beta dispersion* functions from the *Vegan* R package with 999 permutations. Linear mixed models for alpha diversity were adjusted for changes in weight, visceral fat, and interaction between treatment (TRE or non-TRE); time (pre or post) was also included, as were within-subject repeated measures. PERMANOVA models for beta diversity included weight and visceral fat. All statistical analyses were performed in *R* [18].

## 3. Results

### 3.1. TRE Intervention Reduced the Eating Window and Resulted in Weight Loss

Results of the primary and secondary metabolic outcomes have been reported elsewhere [9]. This secondary analysis focuses on the subset of participants from the original trial who had microbiome samples available for analysis (*n* = 16; additional details in the Supplementary Figure S1). Participants in the TRE group successfully reduced their eating window from an average of 15.3 ± 0.8 h at baseline to 9.3 ± 1.7 h during the intervention (mean ± SD, *p* = 3.6 × 10^−6^, Student’s *t*-test). In comparison, the control group’s eating window remained relatively unchanged, with 15.8 ± 1.0 h at baseline and 15.2 ± 1.1 h during the intervention (*p*-value = 0.28). The TRE group experienced significantly greater reductions in weight, visceral fat, and BMI compared to the non-TRE group (*p* < 0.05, Student’s *t*-test, see Table 1).

Of the 16 participants, 5 completed microbiome sample collection at both baseline and post-intervention. The trends observed for changes in eating window and weight loss in this subset were consistent with those in the larger cohort (see Table 2 for specific characteristics of this subset). Stool collection was incorporated into the study after the initiation of the original trial and was presented to participants as optional, which accounts for why only five participants provided stool samples at both time points.

### 3.2. No Observed Impact of TRE on Microbiome Diversity

**Figure 1:**
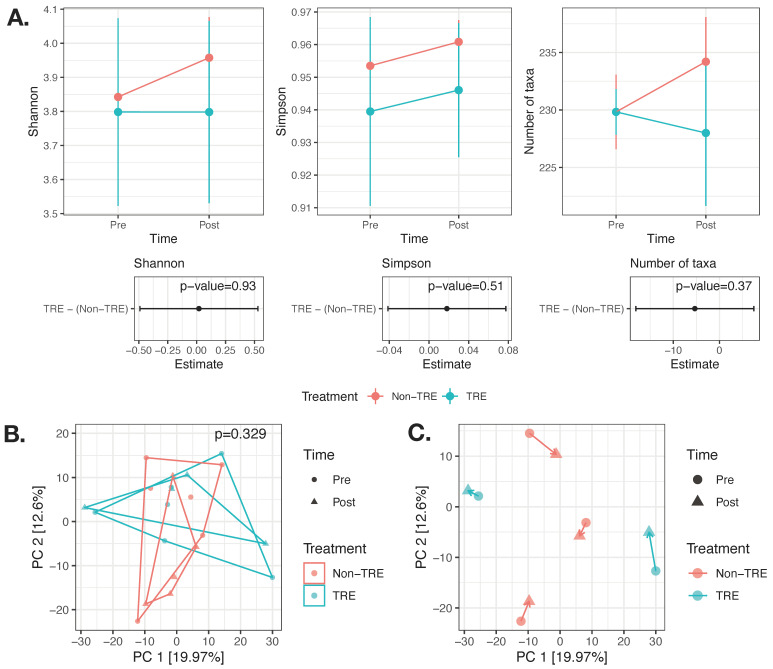
No significant change in alpha or beta diversity after 12 weeks of TRE. (**A**) The effect of the TRE intervention on alpha diversity (Shannon, Simpson, and the number of taxa) was modeled using linear mixed models adjusted for weight change and visceral fat change and accounting for within-subject repeated measures. *n* = 21 samples from 16 participants (8 TRE and 8 Non-TRE). (**B**) Beta diversity modeled using PERMANOVA and adjusted for weight change and visceral fat change and accounting for within-subject repeated measures. *n* = 21 samples from 16 participants (8 TRE and 8 Non-TRE). (**C**) Visualization of the paired subset of samples (*n* = 10 samples from 5 participants) did not reveal clear trends within study arms with respect to directionality of changes over the first principal coordinates. TRE: time-restricted eating; Non-TRE: time-unrestricted eating, PC: principal coordinate.

The effects of the TRE intervention on alpha diversity (Shannon, Simpson, and the number of taxa) were modeled using linear mixed models, adjusted for changes in weight and visceral fat while accounting for within-subject repeated measures. There was no significant effect of the TRE intervention on any of the alpha diversity metrics (Figure 1A).

The effect of the TRE intervention on beta diversity was modeled using PERMANOVA (Figure 1B). No significant difference in beta dispersion was found by treatment (*p* = 0.45) or by time (*p* = 0.77). The TRE intervention did not significantly affect composition (beta diversity), even after controlling for changes in weight and visceral fat (overall *p* = 0.329). Linear models assessing the position along the first five principal component axes, adjusted for weight and visceral fat changes and repeated measures, also showed no association with the TRE intervention.

A visual inspection of the subset of paired samples (*n* = 10 samples from five participants) did not reveal any clear trends within the study arms regarding the directionality of changes over the first principal coordinates (Figure 1C). Given the small sample size (*n* = 5 participants), statistical testing for significant changes within the paired subsample was not conducted.

### 3.3. No Observed Effect of TRE on Microbiome Composition

**Figure 2:**
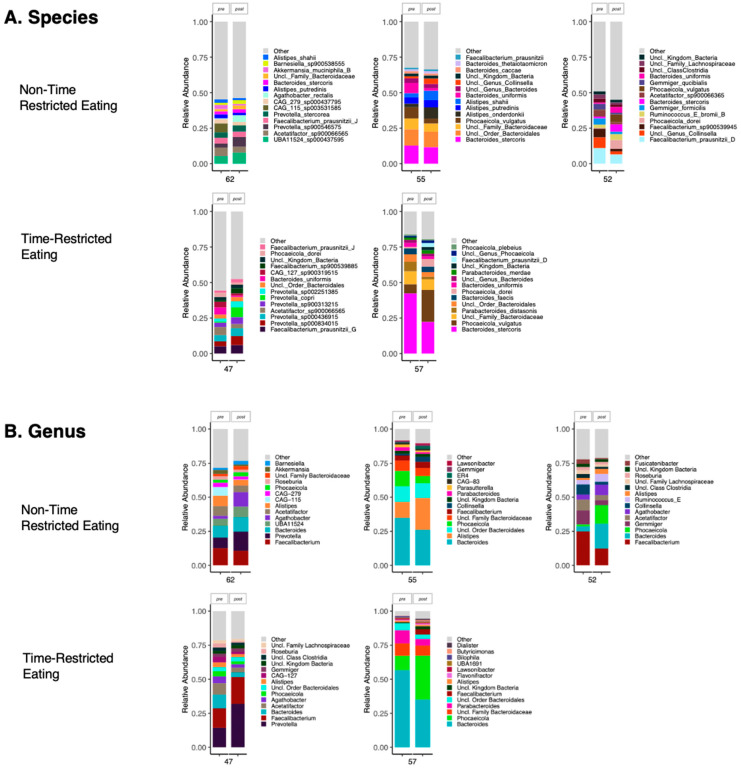
Microbiome composition at the (**A**) species level and (**B**) genus level for participants with paired pre- and post-intervention samples. Five participants had samples available at both pre-intervention and post-intervention time points (*n* = 3 non-TRE and *n* = 2 TRE). The most abundant species and genera are shown for each participant. TRE: time-restricted eating; Non-TRE: time-unrestricted eating.

Species and genus-level microbiome composition were assessed in the five participants who provided samples at both pre- and post-intervention time points (Figure 2). Significant heterogeneity in microbiome composition was observed between participants. No clear trends in species or genus-level changes were detected during either the TRE or non-TRE interventions in this limited subset of samples. Further differential abundance testing was not conducted.

## 4. Discussion

TRE is known to influence body weight, potentially through caloric restriction or other mechanisms related to energy balance. Given the reductions in overall dietary intake and the decreased provision of dietary components to the distal colon during fasting periods, it is plausible to hypothesize that TRE may impact the complex ecosystem and dynamics of the gut microbiome. However, the available microbiome data from human TRE trials are limited and show conflicting results [7,19,20,21]. Therefore, we conducted a secondary analysis of a 12-week randomized controlled trial involving TRE with an 8-h eating window to explore the relationship between TRE and changes in the human gut microbiome. Our analysis did not reveal any significant differences in alpha diversity, beta diversity, or species abundance in the gut microbiome between the TRE intervention group and either the baseline or the non-TRE control group.

Obesity is associated with alterations in the gut microbiome, including reduced microbial diversity [22]. Diet is a key factor influencing microbiome composition [23]. Caloric restriction and subsequent weight loss have been shown to beneficially alter the gut microbiome, often by increasing microbial diversity [24,25,26]. In mouse studies, TRE has been linked to protection against metabolic disease and favorable changes in gut microbiome structure [6,27,28], including increases in metabolically beneficial bacterial groups such as the *Firmicutes* phylum, *Clostridia* class, *Ruminococcaceae* family, and *Roseburia* genus [28]. Based on these findings, we hypothesized that TRE interventions in humans might also yield beneficial effects on gut microbiome composition and diversity, warranting further investigation.

Our findings align with those of a non-randomized, uncontrolled study conducted by Gabel et al. [19]. In that study, the gut microbiome composition of 14 adult participants with obesity was compared between baseline and after 3 months of a TRE intervention with an 8-h eating window. The study did not detect significant changes in microbiome diversity or composition compared to baseline, though the authors suggested that the study may have been underpowered to detect such changes. A trend toward increased diversity following the TRE intervention was observed but was not statistically significant [19].

In contrast, our findings differ from those of Zeb et al., who reported two studies involving healthy men undergoing TRE interventions [7,21]. In the first study, 30 healthy men aged 18–30 years completed either a TRE or non-TRE intervention with an 8-h eating window over 25 days. Stool samples collected at the end of the intervention revealed that the TRE group had a higher abundance of *Prevotella* species (particularly Prevotlla_9) and a lower abundance of *Bacteroides* and *Escherichia-Shigella* compared to the non-TRE group. The authors concluded that TRE alters the microbial composition of the human gut microbiome [7]. In the second study, 80 young healthy men underwent a TRE (8-h eating window) or non-TRE intervention, and stool samples were collected from a subset of participants after 25 days (*n* = 18 post-TRE and *n* = 14 post-non-TRE). TRE significantly increased microbial diversity compared to non-TRE and was associated with enrichment of the *Prevotellaceae* and *Bacteroideaceae* families [21].

It has been proposed that the gut microbiome, particularly the restoration of microbial circadian rhythms, could play a key role in the effectiveness of TRE. In rodents, normal diurnal fluctuations in gut microbiome composition are often disrupted by diet-induced obesity but can be partially restored by TRE [6]. In humans, the composition and function of the gut microbiota also fluctuate throughout the day, influenced by behavioral factors such as eating frequency, time of day, and the duration of overnight fasting [29]. Circadian disturbances, such as jet lag, have been shown to alter microbial composition, and fecal transplants from humans experiencing jet lag to germ-free mice have led to weight gain and glucose intolerance in the recipient mice [30]. Similarly, five days of fasting has been linked to microbiome changes in individuals with metabolic syndrome [31]. However, no prospective human studies have sampled participants undergoing TRE at multiple time points throughout the day. If the gut microbiome is indeed a mechanistic link for TRE’s effectiveness, future studies should explore microbial circadian patterns in addition to diversity and compositional changes.

If the primary dietary change driven by TRE is an overall reduction in caloric intake without altering the composition of the diet as reported by Pavlou et al. [32], it stands to reason that we were unable to detect significant changes in the gut microbiome alpha diversity following TRE. As elegantly explained by Cantu-Jungles and Hamaker, without an external factor introducing new microbial diversity, or dietary shifts that allow previously low-abundance bacteria to proliferate to detectable levels, it is unlikely that we would observe an increase in species diversity in this study [33]. This aligns with the notion that dietary diversity and composition may be key drivers of microbiome changes. This notion may be supported by other natural experiments exploring the changes in the microbiome following, daily fasting during daylight hours throughout the month of Ramadan. Ramadan fasting is associated with weight loss and increased microbial diversity, promoting metabolically favorable taxa [8,34]. Importantly, the cultural eating traditions throughout the month of Ramadan, specifically suhoor and iftar, may mean that fasting during Ramadan reflects changes in the types, quantities, and location of foods consumed, and is, therefore, significantly different from TRE fasting that is not accompanied by a change in dietary pattern. It is important to note that as part of the parent study by Chow et al. study, our participants took food photos using the myCircadianClock (mCC) smartphone application, which allowed documentation of the timing of food consumption and grouping of food types into basic categories [35]. Using this application, the TRE group documented more complete meals and fewer incomplete meals, high-quality snacks, low-quality snacks, and caffeinated beverage intake compared to the control group. Thus, we cannot completely rule out that overall dietary pattern changes alongside TRE may have the potential to impact the gut microbiome composition. It is likely that larger, more tightly controlled studies will be necessary to disentangle the impact of any dietary quality changes from meal timing changes resulting from TRE.

Our analyses have several strengths. We used samples from a previously completed randomized controlled trial of TRE, which included a comparable non-TRE control group [9]. Stool samples were collected from some participants at both pre- and post-intervention time points and analyzed using shotgun metagenomic sequencing. In contrast, many previous studies used only 16S rRNA analysis [7,19,20,21], lacked a control group [19], or collected samples at just one time point [7,21]. Despite limitations in sample availability, the subset of participants in this study exhibited similar weight loss outcomes with TRE as observed in the overall clinical trial, suggesting that this was a representative sample. Additionally, the TRE group reduced their eating window by approximately 6.5 h, while no reduction was observed in the non-TRE group. This meaningful reduction in eating window may be a key factor in TRE effectiveness, which has not been consistently achieved in all TRE trials.

The main limitation of this study is the small sample size and the limited number of paired samples from the same participants at both pre- and post-intervention time points. This may have resulted in the study being underpowered to detect changes in microbial diversity or composition. Future studies should incorporate multiple consecutive samples per time point and longitudinal sampling within participants to improve data collection on the gut microbiome in human trials [36]. Additionally, in our study, stool samples were collected regardless of the time of day and may have missed potential circadian effects on the microbiome. We were also unable to control for caloric intake, fecal moisture, or transit time, as these variables were not measured in the original trial. Fecal water content and stool consistency, as measured by the Bristol Stool Scale, are known to affect alpha diversity. An additional limitation is that our participant population predominantly consisted of women, and results may not be generalizable to men. Our intervention was relatively short, only 12 weeks in duration, and it is possible that a longer duration of TRE could lead to changes in the gut microbiome. Given the small sample size, we did not explore the functional genomic potential of the microbiome samples. It is possible that there could be functional microbiome differences following TRE that are not reflected in microbiome composition. Future dedicated studies of TRE with the microbiome and microbiome function as the primary outcome(s) will be necessary to fully elucidate the effects of TRE on the human gut microbiome.

## 5. Conclusions

In summary, our exploratory study did not demonstrate any significant differences in gut microbiome alpha diversity, beta diversity, or composition in participants completing a TRE intervention compared to controls. The small sample size, combined with stool sampling at only two time points and a limited number of paired pre/post-intervention samples, may have resulted in the study being underpowered to detect meaningful changes. Further investigation in human participants is needed to clarify the potential role of the gut microbiome in mediating the effects of TRE interventions.

## Figures and Tables

**Table 1:** Characteristics of study participants.

Characteristic	Non-TRE, *n* = 8 *	TRE, *n* = 8 *	*p*-Value **
Sex			>0.9
Female	7 (88%)	6 (75%)	
Male	1 (12%)	2 (25%)	
Age	43 (34, 50)	50 (39, 58)	0.4
Ethnicity			>0.9
Hispanic or Latino	1 (12%)	2 (25%)	
Not Hispanic or Latino	7 (88%)	6 (75%)	
Race			0.5
Black or African American	2 (25%)	0 (0%)	
White	6 (75%)	8 (100%)	
Number of samples			>0.9
1	5 (62%)	6 (75%)	
2	3 (38%)	2 (25%)	
Baseline weight (kg)	90 (86, 123)	88 (80, 104)	0.5
Weight change (kg)	−1.75 (−2.05, 0.20)	−2.80 (−4.51, −2.07)	0.023
Baseline BMI (kg/m^2^)	32 (30, 40)	30 (28, 37)	0.6
BMI change (kg/m^2^)	−0.38 (−0.71, 0.11)	−1.02 (−1.44, −0.44)	0.033
Baseline eating window (h)	16.12 (14.88, 16.37)	15.17 (14.97, 15.54)	0.4
Eating window change (h)	−0.68 (−1.21, −0.17)	−6.58 (−7.20, −5.23)	<0.001
Baseline visceral fat (kg)	1.87 (1.44, 2.65)	4.30 (3.38, 4.87)	0.043
Visceral fat change (kg)	0.02 (−0.10, 0.24)	−0.77 (−0.92, −0.02)	0.015

* *n* (%) or Median (interquartile range). ** Fisher’s exact test or Welch Two-Sample *t*-test. TRE: time-restricted eating, Non-TRE: time-unrestricted eating, BMI: body mass index, kg: kilogram, m: meter, h: hours.

**Table 2:** Characteristics of participants with paired pre- and post-intervention microbiome samples.

Characteristic	Non-TRE, *n* = 3 *	TRE, *n* = 2 *
Sex		
Female	3 (100%)	2 (100%)
Age	28 (28, 42)	38 (37, 40)
Ethnicity		
Hispanic or Latino	1 (33%)	1 (50%)
Not Hispanic or Latino	2 (67%)	1 (50%)
Race		
Black or African American	1 (33%)	0 (0%)
White	2 (67%)	2 (100%)
Baseline weight (kg)	92 (89, 113)	84 (78, 90)
Weight change (kg)	−0.10 (−1.05, 1.05)	−1.55 (−1.88, −1.23)
Baseline BMI (kg/m^2^)	33.7 (32.4, 37.3)	32.1 (29.4, 34.8)
BMI change (kg/m^2^)	−0.08 (−0.31, 0.31)	−0.40 (−0.44, −0.35)
Baseline eating window (h)	16.09 (15.19, 16.30)	15.66 (15.32, 15.99)
Eating window change (h)	−0.88 (−1.03, −0.06)	−6.91 (−7.62, −6.20)
Baseline visceral fat (kg)	1.78 (1.30, 2.57)	3.24 (2.47, 4.01)
Visceral fat change (kg)	0.54 (0.28, 0.83)	−0.37 (−0.53, −0.22)

* *n* (%) or Median (interquartile range). TRE: time-restricted eating, Non-TRE: time-unrestricted eating, BMI: body mass index, kg: kilogram, m: meter, h: hours.

## Data Availability

De-identified data will be shared upon request, following review and approval by LSC.

## Supplementary Materials

The following supporting information can be downloaded at: https://www.mdpi.com/article/10.3390/nu17010185/s1, Figure S1: Participant Flow. Adapted from the original publication [9]. TRE: time-restricted eating, Non-TRE: time-unrestricted eating, DXA: dual X-ray absorptiometry, OGTT: oral glucose tolerance test, CGM: continuous glucose monitoring.

## Abbreviations

BMI	body mass index
CGMS	continuous glucose monitoring system
DXA	dual X-ray absorptiometry
HbA1c	hemoglobin A1c
Non-TRE	time-unrestricted eating
OGTT	oral glucose tolerance test
TRE	time-restricted eating
