# Supplementary material for: A Randomized Pilot Study of Time-Restricted Eating Shows Minimal Microbiome Changes
Source: Nutrients. 2025 Jan 4;17(1):185. doi: 10.3390/nu17010185 (PMC11722650; doi:10.3390/nu17010185)
Supplement: Supplementary file 1 [file nutrients-17-00185-s001.zip › nutrients-3327335-supplementary.pdf]

Supplementary Material

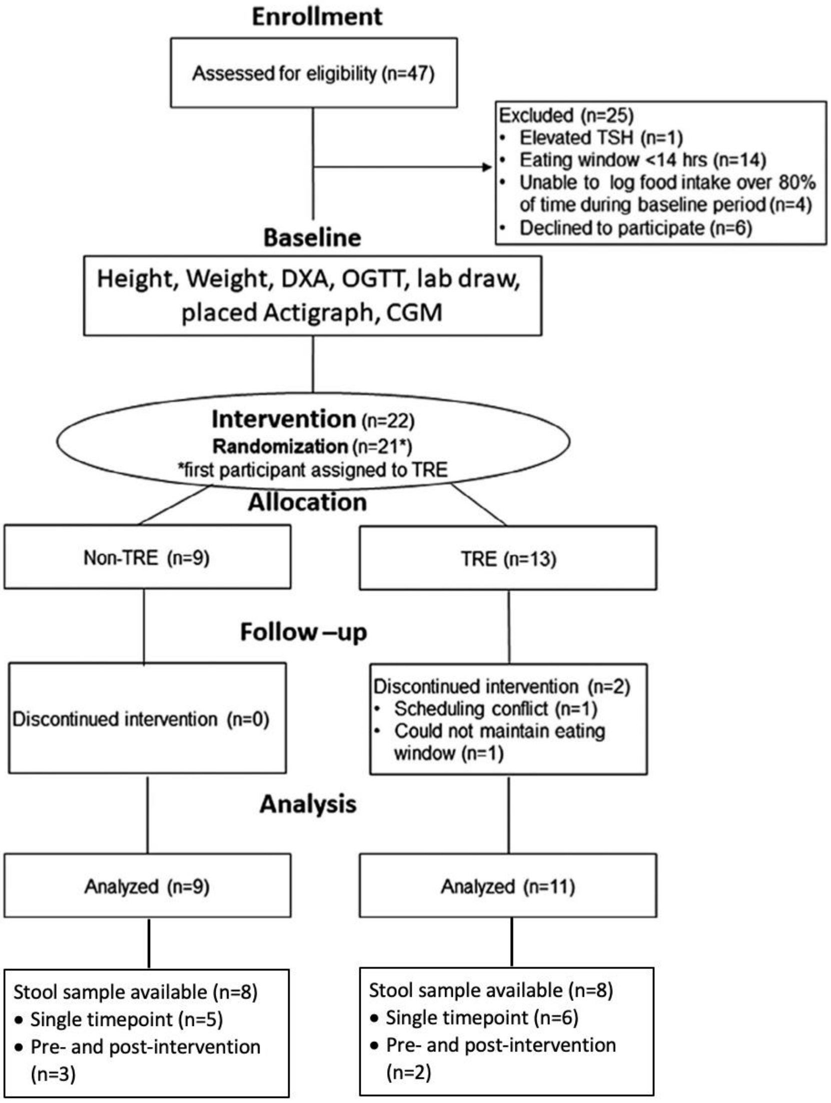

Figure S1. Participant Flow. Adapted from original publication (Chow, *et al*, Obesity 2020). TRE: time-restricted eating, Non-TRE: time unrestricted eating, DXA: dual x-ray absorptiometry, OGTT: oral glucose tolerance test, CGM: continuous glucose monitoring.
